# Risk factors for early-onset colorectal cancer: a population-based case–control study in Ontario, Canada

**DOI:** 10.1007/s10552-021-01456-8

**Published:** 2021-06-13

**Authors:** Vicky C. Chang, Michelle Cotterchio, Prithwish De, Jill Tinmouth

**Affiliations:** 1 Prevention and Cancer Control, Clinical Institutes and Quality Programs, Ontario Health (Cancer Care Ontario), Toronto, ON Canada; 2 Dalla Lana School of Public Health, University of Toronto, Toronto, ON Canada; 3 Department of Medicine, Sunnybrook Health Sciences Centre and University of Toronto, Toronto, ON Canada; 4 Institute of Health Policy, Management and Evaluation, University of Toronto, Toronto, ON Canada

**Keywords:** Early-onset colorectal cancer, Risk factors, Diet, Sugary drinks, Sedentary behavior

## Abstract

**Purpose:**

There has been an alarming increase in colorectal cancer (CRC) incidence among young adults aged < 50 years, and factors driving this upward trend are unknown. This study investigated associations between various medical, lifestyle, and dietary factors and risk of early-onset CRC (EO-CRC).

**Methods:**

A population-based case–control study was conducted in Ontario, Canada during 2018–2019. EO-CRC cases aged 20–49 years (*n* = 175) were identified from the Ontario Cancer Registry; sex- and age group-matched controls (*n* = 253) were recruited through random digit dialing. Data on potential a priori risk factors were collected using a web-based self-reported questionnaire. Odds ratios (OR) and 95% confidence intervals (CI) were estimated using multivariable logistic regression.

**Results:**

Family history of CRC in a first- or second-degree relative (OR 2.37; 95% CI 1.47–3.84), longer sedentary time (≥ 10 vs. < 5 h/day, OR 1.93; 95% CI 1.02–3.65), greater consumption of sugary drinks (≥ 7 vs. < 1 drinks/week, OR 2.99; 95% CI 1.57–5.68), and a more Westernized dietary pattern (quartile 4 vs. 1, OR 1.92; 95% CI 1.01–3.66) were each associated with an increased risk of EO-CRC. Conversely, calcium supplement use (OR 0.53; 95% CI 0.31–0.92), history of allergy or asthma (OR 0.62; 95% CI 0.39–0.98), and greater parity in females (≥ 3 vs. nulliparity, OR 0.29; 95% CI 0.11–0.76) were each associated with a reduced risk.

**Conclusion:**

Modifiable factors, particularly sedentary behavior and unhealthy diet including sugary drink consumption, may be associated with EO-CRC risk. Our findings, if replicated, may help inform prevention strategies targeted at younger persons.

**Supplementary Information:**

The online version contains supplementary material available at 10.1007/s10552-021-01456-8.

## Introduction

Colorectal cancer (CRC) is the third most commonly diagnosed cancer and the second leading cause of cancer deaths globally [1]. In contrast to declining rates among persons aged 50 years or older, which have been largely attributed to screening, CRC incidence rates among young adults (aged < 50) have increased markedly over the past few decades worldwide [2] and in many countries/regions, including Canada [3], the United States [4], Australia [5], and parts of Europe [6, 7] and Eastern Asia [8]. Reasons underlying the alarming rise in early-onset CRC (EO-CRC) are unknown. The increasing prevalence of several traditional CRC risk factors in younger birth cohorts, such as physical inactivity, obesity, and diabetes, has been hypothesized to contribute to the upward trend [9–11]; however, their associations with EO-CRC risk are not consistently supported by the limited epidemiological evidence to date [12].

EO-CRC occurs more commonly in the distal colon and rectum and are often characterized by more advanced stage at diagnosis and aggressive tumor histology [13]. Despite extensive knowledge regarding the etiology of overall CRC (based primarily on older-onset CRC) [14], there is a paucity of literature on risk factors specific to EO-CRC. A recent systematic review and meta-analysis of EO-CRC risk factors identified 20 relevant studies published up to August 2020 and reported significant associations for first-degree family history of CRC, hyperlipidemia, obesity, and alcohol consumption, although analyses for most factors were based on a small number of studies with considerable heterogeneity [15]. One of the first studies investigating EO-CRC risk factors among both men and women was a European hospital-based case–control study conducted in 1985–2009, which reported increased risk associated with family history of CRC, alcohol, and processed meat intake, and no association with physical activity, overweight/obesity, or diabetes [16]. More recently, analyses of prospective cohort data from the Nurses’ Health Study revealed an association between obesity [17], as well as sedentary behavior (assessed as TV viewing time) [18], and increased risk of EO-CRC. In addition, relying on administrative or electronic health record (EHR) data, several retrospective cohort [19–22] and case–control [23, 24] studies have been published recently (since 2019) on EO-CRC risk factors. While most of these studies reported increased EO-CRC risk associated with family history of CRC [19–23], personal history of inflammatory bowel disease (IBD) [19, 21, 23], and/or diabetes [21, 22], they lacked detailed data on modifiable lifestyle factors, such as diet, and potential confounders.

Beyond conventional CRC risk factors, emerging hypotheses suggest the need to also consider “novel” exposures that may be more prevalent among younger generations [11, 12, 25]. These include Westernized diets, processed foods and additives (e.g., high-fructose corn syrup), modulators of the gut microbiome and/or immune system (e.g., antibiotics, allergies), and radiation from medical procedures (e.g., computed tomography [CT] scans) [11, 12, 25, 26], which have yet to be evaluated in epidemiologic studies in relation to EO-CRC risk. Given the increase in EO-CRC incidence and scarcity of knowledge on risk factors for this largely preventable disease, we conducted a study to evaluate associations of various medical, lifestyle, and dietary factors with EO-CRC risk, covering both previously established risk factors for overall CRC as well as a priori hypothesized novel factors.

## Methods

A population-based case–control study was conducted in Ontario, Canada to investigate a wide range of potential risk factors for EO-CRC. The study protocol was approved by the University of Toronto Health Sciences Research Ethics Board.

### Case ascertainment and recruitment

EO-CRC cases were identified through the Ontario Cancer Registry (OCR), a population-based database of all cancer cases in the province of Ontario. Eligible cases were Ontario residents aged 20–49 years at the time of diagnosis with a pathologically confirmed incident invasive colorectal adenocarcinoma (International Classification of Diseases for Oncology, Third Edition [ICD-O-3] topography codes: C18.0, C18.2–C18.9, C19.9, C20.9) between January 2018 and May 2019. Information on tumor microsatellite instability (MSI) status was obtained from pathology reports. MSI status was determined based on immunohistochemistry staining results for DNA mismatch repair proteins (MLH1, MSH2, MSH6, and PMS2), with cases classified as “MSI-high” if they had abnormal staining for any of the four proteins.

Of the 782 eligible EO-CRC cases identified from the OCR, 220 provided opt-in consent to be contacted by our study (by mailing back a signed consent form to Cancer Care Ontario). These 220 patients were then invited by e-mail to participate in our study, of which 175 (80%) completed the online study questionnaire. The median time between diagnosis and questionnaire completion was 10 months (5th–95th percentile: 5–16 months). Compared to all eligible cases who did not participate in the study, participating cases were more likely to be female (58% vs. 43%) but had similar distributions of age at diagnosis (mean: 43 vs. 42 years), stage (III or IV: 60% vs. 64%), and tumor subsite (distal colon or rectum: 76% vs. 74%).

### Control recruitment

Population-based controls, defined as Ontario residents aged 20–49 years (frequency-matched to case estimates by sex and 5-year age group) with no history of CRC, were recruited by the Institute for Social Research at York University (Toronto, Ontario) in 2019 using modified random digit dialing methods. A sampling frame of telephone numbers was constructed using provincial directories and commercially available lists, as well as numbers on either side of listed numbers. Approximately 53,000 randomly selected households were telephoned to identify eligible controls. Of the 1,800 households for which an eligible person was identified, 640 expressed interest and provided their contact information. We then invited these 640 persons by e-mail to participate in our study, of which 253 (40%) completed the online questionnaire.

### Data collection

An invitation letter that included a URL link to access the web-based study questionnaire was e-mailed to all eligible cases and controls who provided initial opt-in consent (cases) or expressed interest in study participation (controls). Generally, non-respondents were followed up by e-mail after 3 weeks, then by telephone 2 weeks later, and a final follow-up e-mail at week 7.

The online questionnaire collected self-reported information on sociodemographics (age, sex, race/ethnicity, birth country, education, income, occupation, rural/urban residence), family history of CRC, personal medical history (type 2 diabetes, IBD, other chronic inflammatory conditions, allergy/asthma), prior medical procedures (sigmoidoscopy/colonoscopy, CT scans), medication use (aspirin/non-steroidal anti-inflammatory drugs [NSAID], laxatives, oral antibiotics), female reproductive history (parity, age at first pregnancy, oral contraceptive [OC] use, menopausal status), body mass index (BMI; weight [kg] divided by height [m] squared), smoking status, secondhand smoke exposure, alcohol consumption, physical activity (adapted from the previously validated Godin-Shephard Leisure-Time Physical Activity Questionnaire, which asked participants to report the number of times they engaged in strenuous [heart beats rapidly, heavy breathing, sweating; e.g., running, jogging, hockey, soccer, squash, basketball, football, cross-country skiing, skating, vigorous swimming, aerobics, vigorous bicycling, spinning, judo] or moderate [slight increase in heart rate and breathing, light sweating; e.g., fast walking, baseball, tennis, easy bicycling, volleyball, badminton, easy swimming, dancing, hiking, downhill skiing, weightlifting] exercise/physical activity for > 15 min during a typical 7-day week [27, 28]), sedentary time (number of hours spent sitting on a typical day at work, school, home, in a car/bus/train, and during leisure time [e.g., watching TV, playing video games, using computer, reading, socializing], averaged over weekdays and weekend days), supplement use (calcium, antacid, vitamin D/cod liver oil, prebiotics, probiotics, folic acid), and consumption of various foods (fruits, vegetables, high-fiber/wholegrain foods, red meat, processed meat, sugary desserts [e.g., candy, chocolate bars, cake, cookies, ice cream], fast food [e.g., burger, fries, taco, pizza, instant ramen noodles], canned food, processed snacks [e.g., chips, crackers, white bread, sugary cereals]), beverages (sugary drinks [e.g., non-diet soft drinks, vitamin drinks, energy drinks, specialty coffee with syrup such as mocha], coffee/tea, water), and sugar substitutes (artificial sweeteners, agave syrup). Dietary intake was assessed as frequency of consumption (daily or weekly) for specified serving sizes as applicable, with detailed examples provided in the questionnaire to aid participant response (e.g., one serving of fruit is: 1 medium-sized fresh fruit, 1/2 cup of chopped, cooked, or canned fruit, 1/4 cup of dried fruit, or 1/2 cup of fruit juice). To ensure pre-diagnosis information was collected, participants were asked to report dietary habits and other lifestyle factors (e.g., alcohol consumption, physical activity, sedentary time) for the time period “2 years ago”. For most other variables, including prior medical diagnoses and procedures and medication or supplement use, participants were asked to only report those occurring at least 2 years before questionnaire completion. Variables are clearly defined in table footnotes.

### Western-like dietary pattern derivation

In addition to examining specific foods/beverages (listed above) as potential EO-CRC risk factors, a composite dietary pattern score was derived as a measure of overall diet quality. This “Western-like” dietary pattern score was derived based on the consumption of six non-beneficial (red meat, processed meat, sugary drinks, sugary desserts, fast food, and processed snacks) and three beneficial (fruits, vegetables, and high-fiber/wholegrain foods) components commonly identified in previous studies [29]. For each non-beneficial component, participants in the first (lowest), second, third, and fourth (highest) quartile of intake (roughly categorized based on distribution among controls) were assigned a value of 0, 1, 2, and 3, respectively. Conversely, for each beneficial component, the quartiles were reverse coded (i.e., 3, 2, 1, and 0 for the first, second, third, and fourth quartile, respectively). The final score was calculated by summing up values across all nine components, with higher scores (range: 0–27) indicating a more Westernized dietary pattern.

### Statistical analysis

Descriptive statistics, including frequencies and proportions, were computed by case–control status for all variables. In general, variables were categorized based on the original categories in the study questionnaire, standard cut-off points (e.g., BMI, physical activity), meaningful cut-offs used in previous studies for ease of interpretation and comparison (e.g., food and beverage consumption), and/or statistical considerations given the distribution among study subjects.

Binary logistic regression analyses were performed to estimate associations between each variable of interest and EO-CRC risk, reported as age- and sex-adjusted and multivariable-adjusted odds ratios (OR), with 95% confidence intervals (CI). Multivariable models were constructed with the main variable of interest and a list of a priori covariates that included age, sex, family history of CRC, aspirin/NSAID use, smoking, physical activity, BMI, alcohol consumption, red/processed meat intake, fruit and vegetable intake, high-fiber food intake, and calcium supplement use. These covariates were chosen as they were established risk/protective factors for overall CRC [14, 30], and they were retained in all models regardless of statistical significance and influence on the effect estimates of other variables. Notably, while diabetes and IBD are also known CRC risk factors, they were not forced into models due to the small proportion of participants reporting each of these conditions (< 5%). Instead, diabetes and IBD, along with several variables not typically considered as CRC risk factors (race/ethnicity, education, income), were evaluated as potential confounders and included in the final model only if their removal resulted in ≥ 15% change in the OR estimate of the main variable.

Analyses were further stratified by sex, and statistical significance of the interaction between sex and each variable was evaluated using the likelihood ratio test. As an exploratory analysis to investigate potential etiologic heterogeneity by anatomical subsite, polytomous logistic regression was used to estimate associations between each variable and CRC tumor subsite-specific risks (proximal colon and distal colon/rectum).

Analyses were performed using SAS, version 9.4 (SAS Institute Inc., Cary, NC). Statistical significance was evaluated at *p* < 0.05, and all tests were two-sided.

## Results

Table 1 presents sociodemographic and clinical characteristics of the 175 EO-CRC cases and 253 controls. The mean ages of cases and controls were 43 and 40 years, respectively. The majority of cases and controls were female (57%), white (81%), had at least a college or university degree (82%), and resided in an urban area for most of their lives (81%). Of cases with available data, 24%, 28%, and 48% had cancer of the proximal colon, distal colon, and rectum, respectively, 11% had MSI-high tumors, and 60% were diagnosed at stage III or IV.

**Table 1:** Sociodemographic and clinical characteristics of early-onset colorectal cancer cases and controls aged 20–49 years, Ontario, Canada, 2018–2019

Characteristics	Cases (*n* = 175)		Controls (*n* = 253)		Age- and sex-adjusted OR^b^ (95% CI)
	*n*^a^ (%)		*n*^a^ (%)		
Age, years					
Mean (SD)	43.1 (5.6)		40.1 (7.9)		N/A
Age group, years					
20–29	6 (3)		36 (14)		N/A
30–34	11 (6)		18 (7)		
35–39	23 (13)		35 (14)		
40–44	38 (22)		69 (27)		
45–49	97 (55)		95 (38)		
Sex					
Male	74 (42)		112 (44)		N/A
Female	101 (58)		141 (56)		
Race/ethnicity					
White	141 (81)		202 (80)		1.00
East/Southeast Asian	16 (9)		16 (6)		1.50 (0.71–3.15)
South Asian	5 (3)		16 (6)		0.57 (0.20–1.66)
Other^c^	13 (7)		17 (7)		1.03 (0.48–2.21)
Country of birth					
Canada	146 (83)		184 (73)		1.00
Outside Canada	29 (17)		69 (27)		0.51 (0.31–0.84)
Highest level of education					
High school graduate or less	32 (18)		46 (19)		1.00
College or university degree	98 (56)		138 (56)		0.97 (0.57–1.66)
Post-graduate degree	45 (26)		64 (26)		0.98 (0.54–1.80)
Annual household income^d^, CAD$					
< $30,000	9 (5)		23 (10)		1.00
$30,000–$69,999	29 (18)		44 (20)		1.56 (0.62–3.93)
$70,000–$100,000	38 (23)		46 (21)		1.91 (0.77–4.70)
> $100,000	88 (54)		108 (49)		1.85 (0.80–4.29)
Unknown (prefer not to answer)	11		32		
Occupation^d^					
Professional^e^	63 (36)		93 (37)		1.00
Managerial and Administrative^f^	33 (19)		41 (16)		1.12 (0.63–1.98)
Sales and Services^g^	25 (14)		34 (13)		1.11 (0.60–2.06)
Clerical^h^	22 (13)		21 (8)		1.46 (0.73–2.94)
Manufacturing, Agriculture, and Trades^i^	17 (10)		26 (10)		1.08 (0.53–2.21)
Student/not employed	7 (4)		27 (11)		0.53 (0.21–1.37)
Other/unspecified	8 (5)		11 (4)		1.01 (0.38–2.67)
Usual residence during most of life					
Urban	140 (81)		202 (81)		1.00
Rural	32 (19)		47 (19)		0.98 (0.59–1.63)
Anatomical subsite^j^					
Proximal colon	41 (24)		N/A		N/A
Distal colon	49 (28)				
Rectum	84 (48)				
MSI status^k^					
Microsatellite stable or MSI-low	122 (89)		N/A		N/A
MSI-high	15 (11)				
Unknown	38				
Stage at diagnosis^l^					
Stage I	28 (19)		N/A		N/A
Stage II	30 (21)				
Stage III	57 (39)				
Stage IV	31 (21)				
Unknown	29				

*CAD* Canadian Dollar; *CI* confidence interval; *MSI* microsatellite instability; *N/A* not applicable; *OR* odds ratio; *SD* standard deviation

^a^Numbers may not sum up to totals due to missing data. An “unknown” category is shown for variables with > 5% missing data

^b^Adjusted for age (continuous, years; age at diagnosis for cases and at questionnaire completion for controls) and sex

^c^Includes Black (*n* = 9), Aboriginal (First Nations, Métis, or Inuit; *n* = 6), and other race/ethnicity (*n* = 15). Numbers broken down by case/control status are not shown due to small cell sizes

^d^Two years ago

^e^Professional occupations in natural and applied sciences, health, education, law and social, community and government services

^f^Management, business, finance and administration occupations

^g^Sales and service occupations, including occupations related to the hospitality and tourism industries

^h^Administrative and office support occupations

^i^Occupations in manufacturing (e.g., metal, glass, chemicals, wood, pulp, textile), agriculture and natural resources (e.g., farming, fishing, forestry), construction, trades, transport and equipment operation

^j^Based on International Classification of Diseases for Oncology, 3rd Edition (ICD-O-3) topography codes (proximal colon: cecum [C18.0], ascending colon [C18.2], hepatic flexure of colon [C18.3], transverse colon [C18.4], and splenic flexure of colon [C18.5]; distal colon: descending colon [C18.6] and sigmoid colon [C18.7]; rectum: rectosigmoid junction [C19.9] and rectum, not otherwise specified [C20.9])

^k^Based on immunohistochemistry staining results for DNA mismatch repair proteins (MLH1, MSH2, MSH6, and PMS2). Cases were considered microsatellite stable or MSI-low if they had normal (intact) nuclear staining for all four proteins, else they were considered MSI-high if they had abnormal (deficient) staining for any of the four proteins

^l^Determined based on multiple sources of staging information, including pathological staging and clinical staging (pathological stage was given priority where available), in accordance with the American Joint Committee on Cancer (AJCC) tumor-node-metastases (TNM) staging system, 8th Edition

Table 2 presents associations between family and personal medical history and EO-CRC risk. Family history of CRC in a first- or second-degree relative was associated with an increased risk of EO-CRC (multivariable-adjusted OR [MVOR] 2.37; 95% CI 1.47–3.84), with a stronger association if at least one relative was diagnosed before 50 years of age (MVOR 3.35; 95% CI 1.25–8.98). Personal history of type 2 diabetes showed a positive but non-significant association with EO-CRC risk (MVOR 1.75; 95% CI 0.57–5.32), while history of allergy/asthma was associated with a statistically significant reduced risk (MVOR 0.62; 95% CI 0.39–0.98), especially when diagnosed before age 10 (MVOR 0.43; 95% CI 0.21–0.88). Furthermore, compared to those who had never had a CT scan, those with 1 or 2 scans had a significantly lower risk (MVOR 0.30; 95% CI 0.16–0.57), whereas those with ≥ 3 CT scans had a suggestive increased risk (MVOR 2.15; 95% CI 0.96–4.81). No associations were observed for ever (vs. never) use of aspirin/NSAID, laxatives, or oral antibiotics; however, oral antibiotic use during childhood alone was associated with a statistically significant lower risk (MVOR 0.29; 95% CI 0.09–0.90). Among females, parity was inversely associated with EO-CRC risk (≥ 3 vs. nulliparity, MVOR 0.29; 95% CI 0.11–0.76; *p*_trend_ = 0.01), whereas positive (albeit non-significant) associations were observed for older age at first pregnancy (≥ 30 vs. < 30 years, MVOR 1.90) and being postmenopausal (MVOR 2.19).

**Table 2:** Associations of family history, medical history, and female reproductive factors with risk of early-onset colorectal cancer, Ontario, Canada, 2018–2019

Variables	Cases (*n* = 175)		Controls (*n* = 253)		Age- and sex-adjusted OR^b^ (95% CI)	Multivariable-adjusted OR^c^ (95% CI)
	*n*^a^ (%)		*n*^a^ (%)			
Family history of CRC^d^						
No	91 (58)		169 (76)		1.00	1.00
Yes	65 (42)		52 (24)		2.20 (1.40–3.45)	2.37 (1.47–3.84)
Age of youngest relative at diagnosis						
< 50 years	12 (8)		9 (4)		2.77 (1.09–7.01)	3.35 (1.25–8.98)
≥ 50 years	53 (34)		43 (19)		2.09 (1.29–3.39)	2.22 (1.33–3.69)
Unknown	19		32			
*Personal medical history*^e^						
Type 2 diabetes						
No	167 (95)		245 (97)		1.00	1.00
Yes	8 (5)		8 (3)		1.32 (0.48–3.61)	1.75 (0.57–5.32)
Chronic inflammatory condition^f^						
No	162 (93)		230 (91)		1.00	1.00
Yes	13 (7)		22 (9)		0.82 (0.40–1.71)	0.89 (0.41–1.92)
Allergy or asthma						
No	122 (71)		151 (60)		1.00	1.00
Yes	51 (29)		99 (40)		0.68 (0.44–1.03)	0.62 (0.39–0.98)
Age at diagnosis						
< 10 years	13 (8)		43 (17)		0.43 (0.22–0.85)	0.43 (0.21–0.88)
10–19 years	23 (13)		34 (14)		0.93 (0.51–1.69)	0.88 (0.46–1.69)
≥ 20 years	15 (9)		22 (9)		0.73 (0.36–1.49)	0.60 (0.28–1.28)
Sigmoidoscopy or colonoscopy						
No	149 (85)		212 (84)		1.00	1.00
Yes	26 (15)		40 (16)		0.78 (0.45–1.35)	0.69 (0.38–1.25)
Total number of CT scans						
Never had a CT scan	133 (79)		165 (70)		1.00	1.00
1–2	15 (9)		58 (24)		0.31 (0.16–0.57)	0.30 (0.16–0.57)
≥ 3	20 (12)		14 (6)		1.99 (0.94–4.23)	2.15 (0.96–4.81)
Unknown	7		16			
Middle and/or lower body CT scan						
No	140 (83)		194 (83)		1.00	1.00
Yes	28 (17)		39 (17)		1.03 (0.59–1.78)	1.02 (0.57–1.82)
Unknown	7		20			
*Medication use*						
Regular aspirin or NSAID use^g^						
Never	119 (68)		180 (71)		1.00	1.00
Ever	56 (32)		73 (29)		1.06 (0.68–1.63)	1.20 (0.75–1.92)
Regular laxative use^g^						
Never	168 (96)		241 (95)		1.00	1.00
Ever	7 (4)		12 (5)		0.79 (0.29–2.12)	0.92 (0.32–2.59)
Oral antibiotic use^h^						
No	139 (79)		191 (75)		1.00	1.00
Yes	36 (21)		62 (25)		0.79 (0.49–1.27)	0.78 (0.47–1.30)
Period of use						
Childhood only (child or teenager)	—^i^		22 (9)		0.30 (0.10–0.91)	0.29 (0.09–0.90)
Adulthood only (age 20s or later)			27 (11)		0.81 (0.42–1.57)	0.86 (0.43–1.70)
Both childhood and adulthood	15 (9)		13 (5)		1.39 (0.63–3.05)	1.42 (0.60–3.37)
*Reproductive history *(*females only*)	(*n* = 101)		(*n* = 141)			
Parity^j^						
0 (nulliparous)	29 (29)		40 (28)		1.00	1.00
1–2	60 (59)		71 (50)		0.65 (0.32–1.30)	0.73 (0.35–1.52)
≥ 3	12 (12)		30 (21)		0.29 (0.12–0.72)	0.29 (0.11–0.76)
Age at first pregnancy (parous women)^j^						
< 30 years	39 (54)		66 (65)		1.00	1.00
≥ 30 years	33 (46)		35 (35)		1.46 (0.78–2.73)	1.90 (0.95–3.79)
Oral contraceptive use^k^						
Never	25 (25)		30 (22)		1.00	1.00
Ever	74 (75)		107 (78)		0.74 (0.40–1.38)	0.65 (0.32–1.33)
Menopausal status^l^						
Premenopausal	84 (83)		131 (93)		1.00	1.00
Postmenopausal	17 (17)		10 (7)		1.90 (0.80–4.53)	2.19 (0.85–5.64)

*CI* confidence interval; *CRC* colorectal cancer; *CT* computed tomography; *NSAID* nonsteroidal anti-inflammatory drug; *OR* odds ratio

^a^Numbers may not sum up to totals due to missing data. An “unknown” category is shown for variables with > 5% missing data

^b^Adjusted for age (continuous, years; age at diagnosis for cases and at questionnaire completion for controls) and sex

^c^Adjusted for age (continuous, years), sex, family history of CRC (no, yes, unknown), regular aspirin/NSAID use (never/ever), smoking (never/ever), physical activity (active, somewhat active, insufficiently active), BMI (continuous, kg/m^2^), alcohol consumption (< once/month, 1–3 times/month, 1–6 times/week, daily), red/processed meat intake (continuous, servings/week), total fruit and vegetable intake (continuous, servings/day), high-fiber food intake (continuous, servings/day), and calcium supplement use (never/ever)

^d^Among any first- or second-degree blood relative

^e^Based only on diagnoses or medical procedures occurring at least 2 years before questionnaire completion

^f^Includes inflammatory bowel disease (Crohn’s disease and ulcerative colitis), celiac disease, rheumatoid arthritis, psoriasis, lupus, and other chronic inflammatory condition (not including allergy or asthma)

^g^Ever taken the medication regularly (at least twice per week for one month or longer) before 2 years ago

^h^Ever used oral antibiotics repeatedly (≥ 2 courses per year) or for an extended period of time (> 1 month) before 2 years ago

^i^Not reported due to small cell counts (*n* < 5 for at least one of the cells)

^j^Based on pregnancies lasting for 6 months or longer. Age at pregnancy was assessed among parous women only (72 cases and 101 controls)

^k^Ever used oral hormonal contraceptives for at least one year before 2 years ago

^l^Females were classified as premenopausal if they had menstrual periods in the last 2 years and postmenopausal if they had stopped menstruating for at least one year before CRC diagnosis (cases) or questionnaire completion (controls) due to natural menopause or surgery to remove the uterus and/or ovaries

Table 3 presents associations between lifestyle factors and EO-CRC risk. While no association was observed for ever (vs. never) smoking, those in the first tertile of pack-years had a significantly elevated risk compared to never smokers (MVOR 1.94; 95% CI 1.04–3.60). Longer sedentary time was associated with a statistically significant increased risk of EO-CRC (≥ 10 vs. < 5 h/day, MVOR 1.93; 95% CI 1.02–3.65; *p*_trend_ = 0.049), whereas BMI at early age 20s and 2 years ago both showed a suggestive inverse association (both *p*_trend_ = 0.06), with respective MVORs of 0.43 (95% CI 0.20–0.90) and 0.59 (95% CI 0.34–1.01) for obesity. Furthermore, despite the lack of statistical significance, longer duration of secondhand smoke exposure and being less physically active tended toward higher risk, while alcohol consumption showed no associations.

**Table 3:** Associations between lifestyle factors and risk of early-onset colorectal cancer, Ontario, Canada, 2018–2019

Variables	Cases (*n* = 175)		Controls (*n* = 253)		Age- and sex-adjusted OR^b^ (95% CI)	Multivariable-adjusted OR^c^ (95% CI)
	*n*^a^ (%)		*n*^a^ (%)			
Smoking status^d^						
Never smoker	110 (63)		172 (68)		1.00	1.00
Ever smoker	65 (37)		81 (32)		1.13 (0.75–1.72)	1.07 (0.68–1.67)
Pack-years of smoking						
Tertile 1 (< 3.5)	33 (19)		27 (11)		1.77 (1.00–3.16)	1.94 (1.04–3.60)
Tertile 2 (3.5–9.9)	12 (7)		27 (11)		0.69 (0.33–1.42)	0.60 (0.27–1.31)
Tertile 3 (≥ 10.0)	20 (11)		27 (11)		0.96 (0.50–1.81)	0.79 (0.40–1.57)
Secondhand smoke exposure^e^						
Two years ago						
Never	115 (70)		167 (70)		1.00	1.00
< 2 h/day	40 (24)		62 (26)		1.17 (0.72–1.91)	1.15 (0.69–1.92)
≥ 2 h/day	9 (5)		9 (4)		1.54 (0.58–4.10)	1.86 (0.64–5.43)
Unknown	11		15			
Childhood and teenage years						
Never	44 (28)		89 (38)		1.00	1.00
< 2 h/day	58 (36)		80 (34)		1.28 (0.77–2.13)	1.22 (0.71–2.11)
≥ 2 h/day	58 (36)		67 (28)		1.35 (0.80–2.29)	1.29 (0.73–2.28)
Unknown	15		17			
Alcohol consumption^f^ (2 years ago)						
Less than once per month	59 (34)		85 (34)		1.00	1.00
1–3 times per month	39 (22)		61 (24)		0.97 (0.57–1.65)	1.05 (0.59–1.85)
1–6 times per week	58 (33)		84 (33)		1.06 (0.65–1.73)	1.01 (0.60–1.70)
Daily	19 (11)		23 (9)		1.24 (0.60–2.55)	1.07 (0.49–2.32)
Physical activity^g^ (2 years ago)						
Active	78 (45)		130 (51)		1.00	1.00
Somewhat active	34 (19)		48 (19)		1.14 (0.67–1.94)	1.16 (0.66–2.02)
Insufficiently active	63 (36)		75 (30)		1.33 (0.85–2.08)	1.46 (0.90–2.38)
Sedentary time^h^ (2 years ago)						
< 5 h/day	41 (23)		73 (29)		1.00	1.00
5 to < 10 h/day	93 (53)		140 (55)		1.20 (0.75–1.93)	1.23 (0.75–2.03)
≥ 10 h/day	41 (23)		40 (16)		1.97 (1.09–3.59)	1.93 (1.02–3.65)
					*p*_trend_^i^ = 0.03	*p*_trend_^i^ = 0.049
Body mass index^j^						
Two years ago						
Normal/underweight	79 (45)		100 (40)		1.00	1.00
Overweight	52 (30)		85 (34)		0.72 (0.45–1.15)	0.57 (0.34–0.94)
Obese	44 (25)		67 (27)		0.74 (0.45–1.22)	0.59 (0.34–1.01)
					*p*_trend_^i^ = 0.26	*p*_trend_^i^ = 0.06
Early age 20s						
Normal/underweight	124 (71)		164 (65)		1.00	1.00
Overweight	39 (22)		53 (21)		1.07 (0.65–1.77)	1.06 (0.63–1.80)
Obese	12 (7)		54 (14)		0.49 (0.24–1.01)	0.43 (0.20–0.90)
					*p*_trend_^i^ = 0.10	*p*_trend_^i^ = 0.06

*CI* confidence interval; *OR* odds ratio

^a^Numbers may not sum up to totals due to missing data. An “unknown” category is shown for variables with > 5% missing data

^b^Adjusted for age (continuous, years; age at diagnosis for cases and at questionnaire completion for controls) and sex

^c^Adjusted for age (continuous, years), sex, family history of CRC (no, yes, unknown), regular aspirin/NSAID use (never/ever), smoking (never/ever), physical activity (active, somewhat active, insufficiently active), BMI (continuous, kg/m^2^), alcohol consumption (< once/month, 1–3 times/month, 1–6 times/week, daily), red/processed meat intake (continuous, servings/week), total fruit and vegetable intake (continuous, servings/day), high-fiber food intake (continuous, servings/day), and calcium supplement use (never/ever)

^d^Ever smoked ≥ 100 cigarettes before 2 years ago. Pack-year was calculated by multiplying the number of packs of cigarettes smoked per day (1 pack = 20 cigarettes) by the number of years smoked

^e^Duration of daily exposure to the tobacco smoke of others at home, work, or public places (averaged over weekdays and weekends)

^f^Frequency of drinking alcoholic beverages (e.g., 12-oz can/bottle of beer, 4-oz glass of wine, 1.5-oz shot of hard liquor) 2 years ago

^g^Defined based on a physical activity score derived using the Godin-Shephard Leisure-Time Physical Activity Questionnaire [27], where weekly frequency (times per week) of strenuous and moderate exercise (2 years ago) was multiplied by 9 and 5, respectively, and summed across activity types to calculate the composite score (active: ≥ 24 units; somewhat active: 14–23 units; insufficiently active: < 14 units)

^h^Average number of hours per day spent sitting at work, at school, at home, in a car/bus/train, and during leisure time (e.g., watching TV, playing video games, using computer, reading, socializing) 2 years ago, calculated as [(5 × number of hours per day sitting on weekdays) + (2 × number of hours per day sitting on weekends)] ÷ 7

^i^*p* value for linear trend calculated by treating the ordinal variable or the median value of each category (where applicable) as a continuous variable in the model, shown only when *p*_trend_ is < 0.10 for at least one of the age- and sex- adjusted and multivariable-adjusted ORs

^j^Calculated by dividing weight (kg) by height (m) squared and classified as normal/underweight (< 25.0 kg/m^2^), overweight (25.0–29.9 kg/m^2^), or obese (≥ 30.0 kg/m^2^)

Table 4 presents associations between dietary factors and EO-CRC risk. Greater consumption of sugary drinks (≥ 7 vs. < 1 drinks/week, MVOR 2.99; 95% CI 1.57–5.68; *p*_trend_ = 0.002), sugary desserts (3–6 vs. < 3 times/week [middle category only], MVOR 2.28; 95% CI 1.28–4.04), and a higher Western-like dietary pattern score (quartile 4 vs. 1, MVOR 1.92; 95% CI 1.01–3.66; *p*_trend_ = 0.047) were associated with elevated risks of EO-CRC. Statistically significant associations were not observed for fruits, vegetables, high-fiber foods, red meat, or processed meat, although greater vegetable consumption showed a tendency toward lower risk (*p*_trend_ = 0.08). In addition, more frequent consumption of fast food (≥ 2 vs. < 1 times/week, MVOR 1.84; 95% CI 0.98–3.46; *p*_trend_ = 0.07) and canned food (≥ 3 vs. < 1 times/week, MVOR 1.70; 95% CI 0.95–3.05; *p*_trend_ = 0.09) showed suggestive associations with increased EO-CRC risk. Of all supplements assessed, calcium was the only one associated with a statistically significant lower risk (ever vs. never use, MVOR 0.53; 95% CI 0.31–0.92).

**Table 4:** Associations between dietary factors and risk of early-onset colorectal cancer, Ontario, Canada, 2018–2019

Variables	Cases (*n* = 175)		Controls (*n* = 253)		Age- and sex-adjusted OR^a^ (95% CI)	Multivariable-adjusted OR^b^ (95% CI)
	*n* (%)		*n* (%)			
*Dietary intake 2 years ago*^c^						
Total fruits and vegetables, servings/day						
< 3	69 (39)		85 (34)		1.00	1.00
3 to < 6	76 (43)		111 (44)		0.69 (0.43–1.08)	0.76 (0.46–1.25)
≥ 6	30 (17)		57 (23)		0.53 (0.30–0.96)	0.58 (0.30–1.13)
					*p*_trend_^d^ = 0.03	*p*_trend_^d^ = 0.11
Fruits, servings/day^e^						
< 1	44 (25)		61 (24)		1.00	1.00
1 to < 3	95 (54)		138 (55)		0.93 (0.57–1.51)	0.96 (0.57–1.60)
≥ 3	36 (21)		54 (21)		0.84 (0.46–1.53)	0.95 (0.49–1.85)
Vegetables, servings/day^f^						
< 1	34 (19)		34 (13)		1.00	1.00
1 to < 3	89 (51)		132 (52)		0.61 (0.35–1.09)	0.56 (0.30–1.03)
≥ 3	52 (30)		87 (34)		0.50 (0.26–0.93)	0.52 (0.26–1.07)
					*p*_trend_^d^ = 0.03	*p*_trend_^d^ = 0.08
High-fiber foods, servings/day^g^						
< 1	69 (39)		93 (37)		1.00	1.00
1 to < 3	74 (42)		114 (45)		0.90 (0.58–1.39)	1.27 (0.77–2.09)
≥ 3	32 (18)		46 (18)		0.94 (0.53–1.66)	1.45 (0.75–2.80)
Red meat, servings/week^h^						
< 2	29 (17)		45 (18)		1.00	1.00
2–4	78 (45)		121 (48)		0.92 (0.52–1.61)	0.82 (0.45–1.51)
≥ 5	68 (39)		87 (34)		1.11 (0.62–1.99)	1.06 (0.56–1.98)
Processed meat, servings/week^h^						
< 1	21 (12)		39 (15)		1.00	1.00
1–2	69 (39)		108 (43)		1.09 (0.58–2.04)	0.96 (0.49–1.88)
≥ 3	85 (49)		106 (42)		1.41 (0.76–2.63)	1.23 (0.62–2.42)
Sugary drinks, drinks/week^i^						
< 1	39 (22)		86 (34)		1.00	1.00
1–6	89 (51)		123 (49)		1.58 (0.98–2.55)	1.86 (1.11–3.13)
≥ 7	47 (27)		44 (17)		2.53 (1.41–4.51)	2.99 (1.57–5.68)
					*p*_trend_^d^ = 0.003	*p*_trend_^d^ = 0.002
Sugary desserts, times/week^j^						
< 3	37 (21)		74 (29)		1.00	1.00
3–6	64 (37)		68 (27)		1.89 (1.11–3.23)	2.28 (1.28–4.04)
≥ 7	74 (42)		111 (44)		1.42 (0.86–2.35)	1.45 (0.86–2.47)
Fast food, times/week^k^						
< 1	26 (15)		55 (22)		1.00	1.00
1	71 (41)		99 (39)		1.57 (0.89–2.77)	1.55 (0.85–2.81)
≥ 2	78 (45)		99 (39)		1.84 (1.03–3.28)	1.84 (0.98–3.46)
					*p*_trend_^d^ = 0.049	*p*_trend_^d^ = 0.07
Canned food, times/week^l^						
< 1	33 (19)		63 (25)		1.00	1.00
1–2	78 (45)		105 (42)		1.34 (0.79–2.27)	1.49 (0.85–2.61)
≥ 3	64 (37)		85 (34)		1.48 (0.86–2.54)	1.70 (0.95–3.05)
					*p*_trend_^d^ = 0.18	*p*_trend_^d^ = 0.09
Processed snacks, times/day^m^						
< 1	116 (66)		183 (72)		1.00	1.00
1 to < 2	39 (22)		48 (19)		1.34 (0.82–2.19)	1.33 (0.79–2.25)
≥ 2	20 (11)		22 (9)		1.43 (0.74–2.79)	1.55 (0.76–3.15)
Coffee or tea, cups/day^n^						
< 1	27 (15)		55 (22)		1.00	1.00
1 to < 3	99 (57)		142 (56)		1.34 (0.78–2.31)	1.43 (0.80–2.54)
≥ 3	49 (28)		56 (22)		1.43 (0.77–2.66)	1.68 (0.85–3.30)
Water, glasses/day						
< 3	59 (34)		75 (30)		1.00	1.00
3 to < 8	94 (54)		136 (54)		0.96 (0.62–1.48)	1.05 (0.65–1.68)
≥ 8	22 (13)		42 (17)		0.76 (0.40–1.45)	0.85 (0.42–1.72)
Artificial sweeteners, times/week						
< 1	116 (66)		173 (68)		1.00	1.00
1–6	31 (18)		53 (21)		0.88 (0.53–1.47)	1.19 (0.68–2.08)
≥ 7	28 (16)		27 (11)		1.46 (0.81–2.63)	1.66 (0.89–3.13)
Agave syrup, times/week						
< 1	166 (95)		226 (89)		1.00	1.00
≥ 1	9 (5)		27 (11)		0.41 (0.19–0.92)	0.45 (0.19–1.04)
Western-like dietary pattern score^o^						
Quartile 1 (0–9)	32 (18)		65 (26)		1.00	1.00
Quartile 2 (10–13)	46 (26)		67 (26)		1.33 (0.75–2.38)	1.46 (0.80–2.67)
Quartile 3 (14–17)	45 (26)		60 (24)		1.58 (0.87–2.85)	1.62 (0.87–3.04)
Quartile 4 (18–27)	52 (30)		61 (24)		1.95 (1.07–3.56)	1.92 (1.01–3.66)
					*p*_trend_^d^ = 0.03	*p*_trend_^d^ = 0.047
*Supplement use before 2 years ago*^p^						
Calcium supplement						
Never	145 (83)		193 (76)		1.00	1.00
Ever	30 (17)		60 (24)		0.58 (0.35–0.98)	0.53 (0.31–0.92)
Antacid						
Never	111 (63)		153 (60)		1.00	1.00
Ever	64 (37)		100 (40)		0.83 (0.55–1.25)	0.87 (0.56–1.36)
Vitamin D/cod liver oil supplement						
Never	114 (65)		146 (58)		1.00	1.00
Ever	61 (35)		107 (42)		0.69 (0.45–1.05)	0.83 (0.52–1.31)
Prebiotic fiber supplement						
Never	138 (79)		196 (77)		1.00	1.00
Ever	37 (21)		57 (23)		0.84 (0.52–1.37)	1.05 (0.62–1.80)
Probiotic supplement						
Never	143 (82)		203 (80)		1.00	1.00
Ever	32 (18)		50 (20)		0.91 (0.54–1.51)	1.09 (0.63–1.89)
Folic acid supplement (females only)	(*n* = 101)		(*n* = 141)			
No	29 (29)		43 (30)		1.00	1.00
Yes	72 (71)		98 (70)		0.72 (0.38–1.35)	0.78 (0.40–1.52)

*CI* confidence interval; *OR* odds ratio

^a^Adjusted for age (continuous, years; age at diagnosis for cases and at questionnaire completion for controls) and sex

^b^Adjusted for age (continuous, years), sex, family history of CRC (no, yes, unknown), regular aspirin/NSAID use (never/ever), smoking (never/ever), physical activity (active, somewhat active, insufficiently active), BMI (continuous, kg/m^2^), alcohol consumption (< once/month, 1–3 times/month, 1–6 times/week, daily), red/processed meat intake (continuous, servings/week), total fruit and vegetable intake (continuous, servings/day), high-fiber food intake (continuous, servings/day), and calcium supplement use (never/ever)

^c^All food and beverage variables are based on usual consumption 2 years before questionnaire completion

^d^*p* value for linear trend calculated by treating the median value of each category as a continuous variable in the model, shown only when *p*_trend_ is < 0.10 for at least one of the age- and sex-adjusted and multivariable-adjusted ORs

^e^Examples for one serving of fruit: 1 medium-sized fresh fruit, 1/2 cup of chopped, cooked, or canned fruit, 1/4 cup of dried fruit, 1/2 cup of fruit juice

^f^Examples for one serving of vegetables: 1 cup of raw leafy vegetables, 1/2 cup of other vegetables (cooked, canned, frozen, or chopped raw), 1/2 cup of vegetable juice

^g^Foods high in fiber, such as wholegrain bread (not white bread), wholegrain or high-fiber breakfast cereal/muesli/bran, brown rice, barley, oats, and legumes (beans, peas, lentils). Examples of one serving of high-fiber food: 1 slice of wholegrain bread, 1/2 cup of cooked or cold high-fiber cereals, 1/2 cup of brown rice or wholegrains

^h^One serving of red/processed meat defined as 2–3 oz or the size of the palm of hand

^i^Sugary drinks such as soft drinks (excluding diet soda), vitamin drinks, energy drinks, and specialty coffee with syrup (e.g., mocha)

^j^Desserts containing sugar, such as candy, chocolate bars, cake, cookies, and ice cream

^k^Includes foods from fast food restaurants (e.g., burger, fries, taco), pizza, and instant meals (e.g., instant ramen noodles)

^l^Any canned foods (e.g., canned corn, canned fruit, canned tomato sauce)

^m^Any processed snack foods such as chips, crackers, white bread, and sugary cereals

^n^Includes both caffeinated and decaffeinated coffee or tea

^o^Composite dietary score derived based on six non-beneficial (red meat, processed meat, sugary drinks, sugary desserts, fast food, and processed snacks) and three beneficial (fruits, vegetables, and high-fiber foods) components. For each non-beneficial component, subjects in the first, second, third, and fourth quartile of intake were assigned a value of 0, 1, 2, and 3, respectively; for each beneficial component, quartiles were reverse coded (i.e., 3, 2, 1, and 0, respectively). The final score was calculated by summing up values across all nine components, with higher scores indicating a more Westernized dietary pattern. The multivariable-adjusted OR was adjusted for all variables listed in footnote b, except for red/processed meat, total fruit and vegetable, and high-fiber food intake

^p^Ever used the supplement regularly (at least once per week for at least one month) before 2 years ago

Sex-stratified analyses revealed similar associations among males and females for most variables, although statistical significance was generally not achieved (Supplementary Tables S1–S3). Notably, greater sugary drink consumption was associated with statistically significantly increased risk of EO-CRC in both males and females (*p*_trend_ < 0.05). Moreover, despite the lack of a significant interaction (*p*_interaction_ = 0.08), coffee/tea consumption was positively associated with EO-CRC risk among males (≥ 3 vs. < 1 cups/day, MVOR 3.08; 95% CI 1.14–8.33; *p*_trend_ = 0.02) but not females (MVOR 0.99; 95% CI 0.40–2.43; *p*_trend_ = 0.97). Another notable sex difference is the tendency toward increased risk for greater red meat consumption among males (≥ 5 vs. < 2 servings/week, MVOR 2.64; 95% CI 0.84–8.33; *p*_trend_ = 0.09), as compared to the non-significant inverse association among females (MVOR 0.68; 95% CI 0.31–1.47; *p*_trend_ = 0.56) (*p*_interaction_ = 0.09).

When analyses were performed by CRC subsite (Supplementary Tables S4–S6), similar patterns of associations were generally observed for proximal colon and distal colon/rectal cancer, with a few exceptions. Notably, calcium supplement use was inversely associated with distal colon/rectal (MVOR 0.40; 95% CI 0.22–0.76), but not proximal colon (MVOR 1.01; 95% CI 0.43–2.37), cancer (*p*_heterogeneity_ = 0.06), and antacid use was differentially associated with risk of proximal colon (MVOR 2.20; 95% CI 1.04–4.63) and distal colon/rectal (MVOR 0.64; 95% CI 0.39–1.06) cancer (*p*_heterogeneity_ = 0.003).

## Discussion

This population-based case–control study found that family history of CRC, longer sedentary time, greater consumption of sugary drinks, and a more Westernized dietary pattern were each associated with a statistically significant increased risk of EO-CRC, whereas history of allergy/asthma, oral antibiotic use during childhood alone, being overweight, calcium supplement use, and greater parity in females were each associated with a statistically significant reduced risk. In addition, more frequent consumption of fast food and canned food showed suggestive associations with increased EO-CRC risk, while greater vegetable consumption tended toward lower risk. Having ≥ 3 CT scans (vs. never) was also associated with a suggestive increased risk, although there was a lack of consistent trend across categories.

While our study confirmed previous findings that family history of CRC is a strong risk factor for EO-CRC [15, 16, 19–23], we did not observe an association for several factors known to affect overall CRC risk, including smoking, alcohol, and aspirin/NSAID. Findings from previous EO-CRC studies regarding these factors have also been inconsistent and inconclusive [15, 16, 19–21, 23, 24, 31], although a recent meta-analysis of EO-CRC risk factors reported a significant association for alcohol consumption (heavy vs. non-drinkers) based on only three studies (pooled relative risk [RR] 1.71; 95% CI 1.62–1.80) [15]. A possible explanation for the general lack of associations may be the long latency required [32, 33] such that established associations with overall (primarily older-onset) CRC may not hold up for EO-CRC. For example, it has been suggested that cigarette smoking plays a stronger role in the initiation of colorectal adenoma, and that its association with increased CRC risk becomes apparent only after a sufficiently long lag period [33]. This hypothesis is supported by the relatively consistent association observed between smoking (as well as alcohol intake) and early-onset colorectal adenoma [34–39]. Meanwhile, an association between smoking (or tobacco use) and EO-CRC risk was only seen in three cohort studies relying on EHR data [19–21], but not in three other case–control studies [23, 24, 31] or the meta-analysis (pooled RR 1.35; 95% CI 0.81–2.25) [15]. Reasons for the increased risk we observed for tertile 1 (vs. never) of smoking pack-years, but not higher tertiles, are unclear and may be a spurious finding. Similarly, only two [16, 20] of five [16, 19–21, 31] previous studies revealed a possible link between alcohol and EO-CRC risk, including a case–control study assessing self-reported consumption [16] and an EHR-based cohort study assessing alcohol-related diagnoses [20]. Furthermore, contrary to our null finding, the only previous study to assess aspirin use in relation to EO-CRC risk (case–control study of US veterans) reported an OR of 0.66 (95% CI 0.52–0.84) [24]. Given the chemoprotective potential of aspirin/NSAID against CRC [40–42], further evaluation of their association with EO-CRC risk is needed.

Despite the lack of statistical significance, we found that personal history of diabetes was associated with a nearly two-fold increase in odds of EO-CRC. While there is convincing evidence of diabetes (and underlying insulin resistance and hyperinsulinemia) as a risk factor for overall CRC [43–45], evidence for EO-CRC remains inconclusive, with some [21, 22, 46], but not all [16, 19, 23, 24], studies reporting a statistically significant association between diabetes and increased EO-CRC risk. Notably, in a Swedish nationwide cohort study that included over 100,000 diabetic individuals diagnosed before age 50, diabetes was associated with a 1.9-fold (95% CI 1.6–2.3) increase in risk of EO-CRC [22]. The small number of diabetic subjects in our and several other studies may have reduced statistical power to detect an association. Additional larger studies are warranted to better evaluate the role of diabetes in EO-CRC etiology, especially given increasing prevalence of diabetes diagnosed in younger persons and potential implications for CRC screening guidelines [22, 47].

Our finding of a positive association between average daily sitting time and EO-CRC risk suggests that sedentary behavior may be a contributor to CRC development among young adults. This corroborates the association observed between prolonged sedentary TV viewing time and increased EO-CRC risk among female nurses in the Nurses’ Health Study [18], although our study further provides evidence for total sedentary time, regardless of activity type, in both males and females. The non-significant association between leisure time physical activity and EO-CRC risk in our study is also comparable to the only other study to have evaluated this association [16]. Meanwhile, an older case–control study among young white men in Los Angeles County reported a suggestive increased risk of EO-CRC associated with lower occupational activity level [31]. Given relatively strong evidence of physical inactivity and sedentary behavior as risk factors for overall CRC [14, 30, 48, 49], and the possibility that these behaviors (and their effects) start early in life [50], there is a need to further assess their roles in EO-CRC etiology.

The rising incidence of EO-CRC has sometimes been attributed to the obesity epidemic [51]. A recent meta-analysis of 7 studies identified obesity as a significant risk factor for EO-CRC (pooled RR 1.54; 95% CI 1.01–2.35); however, cross-sectional studies were included in the analysis and considerable heterogeneity was detected across studies [15]. The suggestive inverse association between BMI and EO-CRC risk in our study contradicts results from the meta-analysis [15], as well as those from the Nurses’ Health Study [17] and analyses of a large EHR database in the US [20, 21], which reported increased risks associated with obesity. Similar to our study, a case–control study of US veterans reported an association between overweight/obesity and reduced risk of EO-CRC [24], while two other case–control studies [16, 23] and a prospective cohort study of African American women [52] reported no associations. While weight loss may be an early symptom of CRC and a possible explanation for the inverse association observed between BMI and EO-CRC risk [24], it is unlikely since a similar association was also observed for BMI at early age 20s in our study. Mechanisms underlying obesity’s role in CRC etiology are complex and likely involve inflammation, insulin resistance, and alterations in adipocytokines, sex hormones, and intestinal microbiota [53, 54]. Future studies examining early-life body fatness (beyond BMI) and related biomarkers may shed light on these conflicting results [12, 55].

Our study is one of the few to examine dietary factors associated with EO-CRC, especially foods/beverages more commonly consumed by recent birth cohorts. Previously, a European case–control study reported that higher processed meat (but not red meat) intake was associated with increased EO-CRC risk, whereas higher intakes of fruits and vegetables were associated with reduced risk [16]. Our results for foods typically known to affect CRC risk (i.e., red/processed meat, fruits/vegetables, fiber/wholegrain) were not statistically significant; however, the suggestive inverse association between vegetable consumption and EO-CRC risk requires verification in larger studies. Likewise, the association between red meat consumption and increased EO-CRC risk in males but not females warrants further investigation, for example, in terms of sex differences in specific meats consumed and cooking method and doneness preferences. More importantly, the association observed for a Western dietary pattern score suggests that poor overall diet quality may be a risk factor for EO-CRC. Western diet, characterized by low-fiber and high-fat/sugar consumption, has been shown to induce inflammation and gut dysbiosis [25, 56, 57] and was associated with increased risk of early-onset colorectal adenoma in a recent cohort study [58]. Our study further suggests associations between processed foods and beverages—hallmarks of a Western diet—and increased EO-CRC risk. In particular, the strong association between sugary drinks and EO-CRC risk supports the hypothesis that high-fructose corn syrup (main sweetener in beverages since the 1980s), along with its negative impacts on insulin sensitivity and gut microbiota, may play a role in EO-CRC etiology [25, 59]. Evidence from animal studies also suggests that high-fructose corn syrup can promote intestinal tumorigenesis and possibly accelerate progression from precursors to CRC [60]. Moreover, although mechanisms remain to be elucidated, the suggestive associations observed for sugary desserts, fast food, and canned foods are likely mediated by the high content of refined sugars, salt, and saturated fats, as well as various chemicals added during the flavoring or processing (e.g., monosodium glutamate, titanium dioxide, synthetic food dyes) and packaging (e.g., bisphenol A) of these foods [25, 61]. In addition, given increasing use of low-calorie sugar substitutes (e.g., sucralose) [62] and their potential role in modifying gut microbiota [59, 63], further research is reasonable as our findings for artificial sweeteners were inconclusive. Our finding of a positive association between coffee/tea consumption and EO-CRC risk in males also requires additional investigation, as these beverages are generally suggested as protective against cancer, although evidence of their associations with overall CRC risk remains inconclusive [30, 64, 65].

To our knowledge, this is the first study to assess dietary supplements in relation to EO-CRC risk. The finding of a protective effect of calcium supplement use is consistent with evidence for overall CRC [30, 66, 67] and may have implications for chemoprevention research [40]. Conversely, calcium intake from foods alone was not associated with EO-CRC risk according to a case–control study that examined dietary intakes of micronutrients [16], possibly suggesting that higher doses of calcium obtained from supplements may be more relevant for EO-CRC prevention, although future studies assessing both dietary and supplemental nutrient intakes are needed. Our results further suggest that the protective effect of calcium supplement may be confined to distal colon/rectal cancer, which is somewhat consistent with the stronger inverse association previously reported for distal compared to proximal colon cancer diagnosed at any age [68]. In addition, although participants were asked to report previous supplement use as > 2 years before questionnaire completion, the association between antacid use (indicated for heartburn/ingestion or bloating) and increased risk of proximal colon, but not distal colon/rectal cancer, may be partly explained by subsite-specific early symptoms for EO-CRC [69].

Beyond diet, antibiotics are also known to influence the gut microbiome [70, 71], which has been implicated in CRC carcinogenesis, possibly through bacterial involvement in nutrient metabolism and direct interaction with gut mucosa [72]. There is concern regarding the increasing prescription of antibiotics in past decades, especially among children and youth, and their possible link to EO-CRC [11, 12, 25]. Recent epidemiological evidence suggests an association between antibiotic use and overall CRC risk, with potential differences by antibiotic type and anatomical subsite [73–76]. Our finding of a lower EO-CRC risk for antibiotic use restricted to childhood and a non-significant increase in risk for use during both childhood and adulthood possibly suggests that use over longer periods may confer increased risk [74]; however, further evaluation with more detailed data (e.g., duration, number of prescriptions) is required.

Another notable epidemiologic shift in recent birth cohorts is the increase in CT scan exposure among pediatric populations [26]. Thus, the suggestive association between having more CT scans (i.e., ≥ 3) and increased EO-CRC risk observed in our study deserves attention in future studies. Previously, a small case–control study reported an association between pelvic irradiation and risk of advanced colorectal neoplasm among young adults [77], and an Australian cohort study reported excess risks of gastrointestinal malignancies among persons exposed to a greater number of CT scans during childhood or adolescence [78]. Notably, the increased risk associated with having 3 or more CT scans but a lower risk with only 1 or 2 scans in our study may be indicative of a non-linear dose–response effect frequently seen for low-dose ionizing radiation [79, 80], although further investigation is needed given the relatively small numbers and absence of information on indication of CT scan.

Allergic conditions have been associated with reduced risks of certain cancers [81]; however, the association with CRC remains inconsistent [82–84]. Our study found an association between history of allergy/asthma, especially earlier age at onset (and hence longer duration since onset), and reduced EO-CRC risk. Underlying mechanisms are unclear but may involve enhanced tumor immunosurveillance and immunoglobulin E-mediated immune responses against colorectal neoplasia [82, 85].

Evidence regarding the associations of reproductive factors with CRC risk is inconclusive [86–88], with the exception of exogenous hormones (OC and hormone replacement therapy [HRT]) as potential protective factors [89, 90]. While OC use was not associated with EO-CRC risk in our study (and HRT was not assessed due to small numbers in this largely premenopausal sample), the reduced risk associated with parity may be partly explained by changes in endogenous sex hormones (e.g., estrogen, prolactin) during pregnancy [91] and warrants additional investigation according to menopausal status.

This study is one of the very few to investigate risk factors for EO-CRC and is the most comprehensive one to date. Strengths of the study included the population-based design, assessment of a wide range of traditional CRC risk factors, as well as novel medical, lifestyle, and dietary factors, and use of a web-based questionnaire to facilitate quality control of data collection. Our study also had several limitations. First, low response rates may have introduced selection bias; however, participating cases were not markedly different from eligible non-participating EO-CRC cases identified from the OCR in terms of age, stage, and cancer subsite. Participating controls also had similar distributions of major lifestyle factors (e.g., smoking, obesity) compared to those of the general young adult population in Ontario [92]. Second, survivor bias is possible though unlikely since most cases were recruited within several months of diagnosis. Additionally, the possibility of recall bias, measurement error, and type I error could not be ruled out. Finally, due to the relatively small sample size, our study may have been underpowered to detect modest associations, and sex- and subsite-specific analyses should be interpreted with caution.

In conclusion, this study provides novel findings on a range of factors possibly associated with EO-CRC risk. Several modifiable factors, particularly sedentary behavior and unhealthy diet characterized by sugary drinks and fast food, emerged as potential risk factors for EO-CRC, while calcium supplement use may be associated with reduced risk. Findings from this study represent early steps in understanding the etiology of CRC among younger persons and warrant confirmation in large prospective studies with long follow-up so that prevention and screening strategies may be targeted at this subpopulation.

## Supplementary Information

Below is the link to the electronic supplementary material.

Supplementary file1 (PDF 351 kb)

## Data Availability

The data described in this manuscript are not publicly available due to privacy and ethical restrictions.

## Code availability

Available upon reasonable request.
