# Supplementary material for: Risk factors for early-onset colorectal cancer: a population-based case–control study in Ontario, Canada
Source: Cancer Causes Control. 2021 Jun 13;32(10):1063–83. doi: 10.1007/s10552-021-01456-8 (PMC8416813; doi:10.1007/s10552-021-01456-8)
Supplement: Supplementary file 1 — Supplementary file1 (PDF 351 kb) [file 10552_2021_1456_MOESM1_ESM.pdf]

Supplementary Material

Risk factors for early-onset colorectal cancer: a population-based case–control study in Ontario, Canada

Vicky C. Chang[1,2]*, Michelle Cotterchio[1,2], Prithwish De[1], and Jill Tinmouth[1,3,4]

[1] Prevention and Cancer Control, Clinical Institutes and Quality Programs, Ontario Health (Cancer Care Ontario), Toronto, ON, Canada

[2] Dalla Lana School of Public Health, University of Toronto, Toronto, ON, Canada

[3] Department of Medicine, Sunnybrook Health Sciences Centre and University of Toronto, Toronto, ON, Canada

[4] Institute of Health Policy, Management and Evaluation, University of Toronto, Toronto, ON, Canada

***Corresponding author:** Vicky C. Chang (e-mail: vickycd.chang@mail.utoronto.ca).

Supplementary Table S1. Sex-specific multivariable-adjusted odds ratios for the associations of family history and medical history with risk of early-onset colorectal cancer, Ontario, Canada, 2018–2019

Variables	**Males** (74 cases; 112 controls) MVOR[a] (95% CI)	**Females** (101 cases; 141 controls) MVOR[a] (95% CI)	$p_{interaction}$[b]
Family history of CRC[c]			0.38
No	1.00	1.00	
Yes	3.04 (1.46–6.32)	1.99 (1.07–3.70)	
Age of youngest relative at diagnosis			0.18
<50 years	1.42 (0.22–9.26)	4.46 (1.36–14.6)	
≥50 years	3.37 (1.56–7.31)	1.61 (0.83–3.15)	
Personal medical history[d]			
Type 2 diabetes			0.64
No	1.00	1.00	
Yes	2.17 (0.51–9.29)	1.28 (0.23–7.13)	
Chronic inflammatory condition[e]			0.31
No	1.00	1.00	
Yes	0.53 (0.15–1.91)	1.23 (0.46–3.27)	
Allergy or asthma			0.18
No	1.00	1.00	
Yes	0.84 (0.44–1.60)	0.45 (0.23–0.87)	
Age at diagnosis			0.45
<10 years	0.55 (0.23–1.34)	0.30 (0.09–1.01)	
10–19 years	1.23 (0.48–3.14)	0.63 (0.25–1.58)	
≥20 years	1.12 (0.33–3.78)	0.39 (0.15–1.05)	
Sigmoidoscopy or colonoscopy			0.68
No	1.00	1.00	
Yes	0.80 (0.32–2.03)	0.63 (0.29–1.34)	
Total number of CT scans			0.06
Never had a CT scan	1.00	1.00	
1–2	0.71 (0.28–1.81)	0.16 (0.06–0.39)	
≥3	3.36 (0.98–11.5)	1.47 (0.51–4.24)	
Middle and/or lower body CT scan			0.38
No	1.00	1.00	
Yes	1.41 (0.56–3.57)	0.83 (0.40–1.74)	
Medication use			
Regular aspirin or NSAID use[f]			0.73
Never	1.00	1.00	
Ever	1.34 (0.62–2.88)	1.14 (0.64–2.02)	
Regular laxative use[f]			
Never	—[g]	1.00	
Ever		0.60 (0.19–1.93)	
Oral antibiotic use[h]			0.86
Never	1.00	1.00	
Ever	0.83 (0.37–1.89)	0.76 (0.40–1.43)	

Period of oral antibiotic use			0.89
Childhood only (child or teenager)	0.17 (0.02–1.52)	0.37 (0.09–1.43)	
Adulthood only (age 20s or later)	0.97 (0.32–2.95)	0.79 (0.33–1.88)	
Both childhood and adulthood	1.87 (0.43–8.09)	1.24 (0.44–3.50)	

CI confidence interval; *CRC* colorectal cancer; *CT* computed tomography; *MVOR* multivariable-adjusted odds ratio; *NSAID* nonsteroidal anti-inflammatory drug.

[a]Adjusted for age (continuous, years), family history of CRC (no, yes, unknown), regular aspirin/NSAID use (never/ever), smoking (never/ever), physical activity (active, somewhat active, insufficiently active), BMI (continuous, kg/m^2), alcohol consumption (<once/month, 1–3 times/month, 1–6 times/week, daily), red/processed meat intake (continuous, servings/week), total fruit and vegetable intake (continuous, servings/day), high-fiber food intake (continuous, servings/day), and calcium supplement use (never/ever).

[b]*p* value for interaction between sex and each variable, calculated using the likelihood ratio test.

[c]Among any first- or second-degree blood relative.

[d]Based only on diagnoses or medical procedures occurring at least 2 years before questionnaire completion.

[e]Includes inflammatory bowel disease (Crohn’s disease and ulcerative colitis), celiac disease, rheumatoid arthritis, psoriasis, lupus, and other chronic inflammatory condition (not including allergy or asthma).

[f]Ever taken the medication regularly (at least twice per week for one month or longer) before 2 years ago.

[g]Association not evaluated due to at least one empty cell (i.e., none reporting ever use).

[h]Ever used oral antibiotics repeatedly (≥2 courses per year) or for an extended period of time (>1 month) before 2 years ago.

Supplementary Table S2. Sex-specific multivariable-adjusted odds ratios for the associations between lifestyle factors and risk of early-onset colorectal cancer, Ontario, Canada, 2018–2019

Variables	Males (74 cases; 112 controls) MVOR[a] (95% CI)	Females (101 cases; 141 controls) MVOR[a] (95% CI)	$p_{interaction}$[b]
Smoking status[c]			0.88
Never smoker	1.00	1.00	
Ever smoker	1.10 (0.57–2.14)	1.04 (0.57–1.87)	
Pack-years of smoking			0.45
Tertile 1 (<3.5)	2.84 (1.11–7.26)	1.43 (0.63–3.24)	
Tertile 2 (3.5–9.9)	0.68 (0.21–2.23)	0.55 (0.20–1.52)	
Tertile 3 (≥10.0)	0.55 (0.20–1.49)	1.10 (0.43–2.84)	
Secondhand smoke exposure[d]			
Two years ago			0.27
Never	1.00	1.00	
<2 hours/day	1.02 (0.49–2.12)	1.28 (0.63–2.58)	
≥2 hours/day	0.86 (0.16–4.59)	3.15 (0.78–12.7)	
Childhood and teenage years			0.87
Never	1.00	1.00	
<2 hours/day	1.62 (0.72–3.61)	0.99 (0.48–2.05)	
≥2 hours/day	1.23 (0.53–2.84)	1.35 (0.64–2.88)	
Alcohol consumption[e] (2 years ago)			0.57
Less than once per month	1.00	1.00	
1–3 times per month	0.96 (0.36–2.55)	1.10 (0.55–2.20)	
1–6 times per week	1.36 (0.62–2.99)	0.77 (0.39–1.54)	
Daily	1.05 (0.39–2.82)	1.43 (0.36–5.71)	
Physical activity[f] (2 years ago)			0.33
Active	1.00	1.00	
Somewhat active	1.41 (0.64–3.09)	0.96 (0.43–2.15)	
Insufficiently active	1.91 (0.91–3.97)	1.20 (0.64–2.25)	
	p_{trend}[g] = 0.08	p_{trend}[g] = 0.58	
Sedentary time[h] (2 years ago)			0.52
<5 hours/day	1.00	1.00	
5 to <10 hours/day	1.76 (0.81–3.86)	0.96 (0.50–1.83)	
≥10 hours/day	2.35 (0.95–5.81)	1.70 (0.69–4.17)	
	p_{trend}[g] = 0.07	p_{trend}[g] = 0.33	
Body mass index[i]			
Two years ago			0.56
Normal/underweight	1.00	1.00	
Overweight	0.87 (0.41–1.83)	0.40 (0.20–0.79)	
Obese	0.71 (0.32–1.60)	0.53 (0.26–1.09)	
	p_{trend}[g] = 0.41	p_{trend}[g] = 0.07	
Early age 20s			0.56
Normal/underweight	1.00	1.00	
Overweight	1.45 (0.73–2.86)	0.65 (0.28–1.54)	
Obese	0.44 (0.15–1.28)	0.43 (0.15–1.23)	
	p_{trend}[g] = 0.33	p_{trend}[g] = 0.08	

CI confidence interval; *MVOR* multivariable-adjusted odds ratio.

[a]Adjusted for age (continuous, years), family history of CRC (no, yes, unknown), regular aspirin/NSAID use (never/ever), smoking (never/ever), physical activity (active, somewhat active, insufficiently active), BMI (continuous, kg/m^2), alcohol consumption (<once/month, 1–3 times/month, 1–6 times/week, daily), red/processed meat intake (continuous, servings/week), total fruit and vegetable intake (continuous, servings/day), high-fiber food intake (continuous, servings/day), and calcium supplement use (never/ever).

[b]*p* value for interaction between sex and each variable (treated as ordinal if applicable), calculated using the likelihood ratio test.

[c]Ever smoked ≥100 cigarettes before 2 years ago. Pack-year was calculated by multiplying the number of packs of cigarettes smoked per day (1 pack = 20 cigarettes) by the number of years smoked.

[d]Duration of daily exposure to the tobacco smoke of others at home, work, or public places (averaged over weekdays and weekends).

[e]Frequency of drinking alcoholic beverages (e.g., 12-oz can/bottle of beer, 4-oz glass of wine, 1.5-oz shot of hard liquor) 2 years ago.

[f]Defined based on a physical activity score derived using the Godin-Shephard Leisure-Time Physical Activity Questionnaire [27], where weekly frequency (times per week) of strenuous and moderate exercise (2 years ago) was multiplied by 9 and 5, respectively, and summed across activity types to calculate the composite score (active: ≥24 units; somewhat active: 14–23 units; insufficiently active: <14 units).

[g]*p* value for linear trend calculated by treating the ordinal variable or the median value of each category (where applicable) as a continuous variable in the model, shown only when p_{trend} is < 0.10 for at least one of the sexes.

[h]Average number of hours per day spent sitting at work, at school, at home, in a car/bus/train, and during leisure time (e.g., watching TV, playing video games, using computer, reading, socializing) 2 years ago, calculated as [(5 × number of hours per day sitting on weekdays) + (2 × number of hours per day sitting on weekends)] ÷ 7.

[i]Calculated by dividing weight (kg) by height (m) squared and classified as normal/underweight (<25.0 kg/m^2), overweight (25.0–29.9 kg/m^2), or obese (≥30.0 kg/m^2).

Supplementary Table S3. Sex-specific multivariable-adjusted odds ratios for the associations between lifestyle factors and risk of early-onset colorectal cancer, Ontario, Canada, 2018–2019

Variables	Males (74 cases; 112 controls) MVOR[a] (95% CI)	Females (101 cases; 141 controls) MVOR[a] (95% CI)	$p_{interaction}$[b]
Dietary intake 2 years ago[c]			
Total fruits and vegetables, servings/day			0.37
<3	1.00	1.00	
3 to <6	0.75 (0.38–1.47)	0.74 (0.37–1.49)	
≥6	0.98 (0.33–2.88)	0.47 (0.21–1.07)	
	p_{trend}[d]= 0.71	p_{trend}[d] = 0.07	
Fruits, servings/day[e]			0.34
<1	1.00	1.00	
1 to <3	0.96 (0.48–1.92)	0.91 (0.43–1.94)	
≥3	1.47 (0.53–4.12)	0.75 (0.31–1.80)	
Vegetables, servings/day[f]			0.72
<1	1.00	1.00	
1 to <3	0.68 (0.33–1.42)	0.34 (0.11–1.04)	
≥3	0.62 (0.24–1.63)	0.34 (0.11–1.06)	
High-fiber foods, servings/day[g]			0.74
<1	1.00	1.00	
1 to <3	1.36 (0.69–2.68)	1.20 (0.61–2.37)	
≥3	1.18 (0.41–3.37)	1.56 (0.69–3.53)	
Red meat, servings/week[h]			0.09
<2	1.00	1.00	
2–4	1.82 (0.59–5.65)	0.60 (0.29–1.25)	
≥5	2.64 (0.84–8.33)	0.68 (0.31–1.47)	
	p_{trend}[d] = 0.09	p_{trend}[d] = 0.56	
Processed meat, servings/week[h]			0.58
<1	1.00	1.00	
1–2	1.38 (0.42–4.61)	0.82 (0.37–1.82)	
≥3	1.82 (0.57–5.85)	1.01 (0.45–2.30)	
Sugary drinks, drinks/week[i]			0.84
<1	1.00	1.00	
1–6	1.54 (0.67–3.55)	2.10 (1.11–4.00)	
≥7	3.00 (1.17–7.69)	2.77 (1.18–6.49)	
	p_{trend}[d] = 0.02	p_{trend}[d] = 0.04	
Sugary desserts, times/week[j]			0.86
<3	1.00	1.00	
3–6	2.50 (1.04–6.00)	2.13 (1.01–4.48)	
≥7	1.44 (0.64–3.23)	1.47 (0.73–2.96)	
Fast food, times/week[k]			0.82
<1	1.00	1.00	
1	2.27 (0.71–7.28)	1.32 (0.66–2.67)	
≥2	2.34 (0.74–7.42)	1.76 (0.81–3.84)	

Canned food, times/week[l]			0.86
<1	1.00	1.00	
1–2	1.84 (0.79–4.31)	1.27 (0.61–2.65)	
≥3	1.81 (0.75–4.34)	1.64 (0.75–3.57)	
Processed snacks, times/day[m]			0.60
<1	1.00	1.00	
1 to <2	1.16 (0.53–2.58)	1.48 (0.74–2.98)	
≥2	1.28 (0.42–3.87)	1.77 (0.71–4.38)	
Coffee or tea, cups/day[n]			0.08
<1	1.00	1.00	
1 to <3	2.25 (0.94–5.40)	0.96 (0.44–2.09)	
≥3	3.08 (1.14–8.33)	0.99 (0.40–2.43)	
	p_{trend}[d] = 0.02	p_{trend}[d] = 0.97	
Water, glasses/day			0.50
<3	1.00	1.00	
3 to <8	0.76 (0.39–1.49)	1.42 (0.74–2.73)	
≥8	0.70 (0.20–2.44)	1.04 (0.45–2.40)	
Artificial sweeteners, times/week			0.64
<1	1.00	1.00	
1–6	1.19 (0.52–2.73)	1.19 (0.58–2.46)	
≥7	1.38 (0.50–3.79)	1.87 (0.84–4.16)	
Agave syrup, times/week			0.59
<1	1.00	1.00	
≥1	0.31 (0.06–1.54)	0.52 (0.19–1.42)	
Western-like dietary pattern score[o]			0.90
Quartile 1 (0–9)	1.00	1.00	
Quartile 2 (10–13)	3.56 (1.10–11.5)	1.00 (0.49–2.06)	
Quartile 3 (14–17)	2.05 (0.67–6.26)	1.61 (0.74–3.50)	
Quartile 4 (18–27)	2.76 (0.96–7.95)	1.77 (0.72–4.32)	
Supplement use before 2 years ago[p]			
Calcium supplement			0.47
Never	1.00	1.00	
Ever	0.36 (0.10–1.23)	0.59 (0.32–1.08)	
Antacid			0.71
Never	1.00	1.00	
Ever	0.96 (0.50–1.83)	0.81 (0.45–1.46)	
Vitamin D/cod liver oil supplement			0.87
Never	1.00	1.00	
Ever	0.79 (0.38–1.63)	0.85 (0.48–1.51)	
Prebiotic fiber supplement			0.92
Never	1.00	1.00	
Ever	1.01 (0.40–2.54)	1.07 (0.56–2.05)	
Probiotic supplement			0.64
Never	1.00	1.00	
Ever	0.89 (0.32–2.47)	1.18 (0.62–2.25)	

CI confidence interval; *MVOR* multivariable-adjusted odds ratio.

[a]Adjusted for age (continuous, years), family history of CRC (no, yes, unknown), regular aspirin/NSAID use (never/ever), smoking (never/ever), physical activity (active, somewhat active, insufficiently active), BMI (continuous,

kg/m^2), alcohol consumption (<once/month, 1–3 times/month, 1–6 times/week, daily), red/processed meat intake (continuous, servings/week), total fruit and vegetable intake (continuous, servings/day), high-fiber food intake (continuous, servings/day), and calcium supplement use (never/ever).

[b]*p* value for interaction between sex and each variable (treated as ordinal if applicable), calculated using the likelihood ratio test.

[c]All food and beverage variables are based on usual consumption 2 years before questionnaire completion.

[d]*p* value for linear trend calculated by treating the median value of each category as a continuous variable in the model, shown only when p_{trend} is < 0.10 for at least one of the sexes.

[e]Examples for one serving of fruit: 1 medium-sized fresh fruit, 1/2 cup of chopped, cooked, or canned fruit, 1/4 cup of dried fruit, 1/2 cup of fruit juice.

[f]Examples for one serving of vegetables: 1 cup of raw leafy vegetables, 1/2 cup of other vegetables (cooked, canned, frozen, or chopped raw), 1/2 cup of vegetable juice.

[g]Foods high in fiber, such as wholegrain bread (not white bread), wholegrain or high-fiber breakfast cereal/muesli/bran, brown rice, barley, oats, and legumes (beans, peas, lentils). Examples of one serving of high-fiber food: 1 slice of wholegrain bread, 1/2 cup of cooked or cold high-fiber cereals, 1/2 cup of brown rice or wholegrains.

[h]One serving of red/processed meat defined as 2–3 oz or the size of the palm of hand.

[i]Sugary drinks such as soft drinks (excluding diet soda), vitamin drinks, energy drinks, and specialty coffee with syrup (e.g., mocha).

[j]Desserts containing sugar, such as candy, chocolate bars, cake, cookies, and ice cream.

[k]Includes foods from fast food restaurants (e.g., burger, fries, taco), pizza, and instant meals (e.g., instant ramen noodles).

[l]Any canned foods (e.g., canned corn, canned fruit, canned tomato sauce).

[m]Any processed snack foods such as chips, crackers, white bread, and sugary cereals.

[n]Includes both caffeinated and decaffeinated coffee or tea.

[o]Composite dietary score derived based on 6 non-beneficial (red meat, processed meat, sugary drinks, sugary desserts, fast food, and processed snacks) and 3 beneficial (fruits, vegetables, and high-fiber foods) components. For each non-beneficial component, subjects in the first, second, third, and fourth quartile of intake were assigned a value of 0, 1, 2, and 3, respectively; for each beneficial component, quartiles were reverse coded (i.e., 3, 2, 1, and 0, respectively). The final score was calculated by summing up values across all 9 components, with higher scores indicating a more Westernized dietary pattern. The MVOR was adjusted for all variables listed in footnote a, except for red/processed meat, total fruit and vegetable, and high-fiber food intake.

[p]Ever used the supplement regularly (at least once per week for at least one month) before 2 years ago.

Supplementary Table S4. Multivariable-adjusted odds ratios for the associations of family history, medical history, and female reproductive factors with risk of early-onset colorectal cancer according to anatomical subsite,[a] Ontario, Canada, 2018–2019

**Variables**	**Proximal colon (*n* = 41) MVOR[b] (95% CI)**	**Distal colon or rectum (*n* = 133) MVOR[b] (95% CI)**	$p_{heterogeneity}$[c]
Family history of CRC[d]			0.05
No	1.00	1.00	
Yes	1.23 (0.55–2.76)	2.87 (1.69–4.88)	
Age of youngest relative at diagnosis			0.13
<50 years	1.16 (0.20–6.72)	3.94 (1.35–11.5)	
≥50 years	1.25 (0.53–2.92)	2.72 (1.56–4.73)	
Personal medical history[e]			
Type 2 diabetes			0.81
No	1.00	1.00	
Yes	1.92 (0.40–9.11)	1.57 (0.44–5.57)	
Chronic inflammatory condition[f]			0.50
No	1.00	1.00	
Yes	0.61 (0.16–2.33)	1.00 (0.43–2.34)	
Allergy or asthma			0.44
No	1.00	1.00	
Yes	0.47 (0.20–1.08)	0.66 (0.40–1.10)	
Age at diagnosis			0.87
<10 years	0.28 (0.06–1.26)	0.45 (0.20–0.98)	
10–19 years	0.75 (0.25–2.27)	0.93 (0.46–1.90)	
≥20 years	0.43 (0.11–1.67)	0.69 (0.30–1.56)	
Sigmoidoscopy or colonoscopy			0.76
No	1.00	1.00	
Yes	0.78 (0.29–2.10)	0.66 (0.34–1.28)	
Total number of CT scans			0.17
Never had a CT scan	1.00	1.00	
1–2	0.37 (0.12–1.15)	0.25 (0.12–0.52)	
≥3	4.15 (1.47–11.7)	1.51 (0.60–3.77)	
Middle and/or lower body CT scan			0.06
No	1.00	1.00	
Yes	1.80 (0.78–4.18)	0.73 (0.37–1.44)	
Medication use			
Regular aspirin or NSAID use[g]			0.16
Never	1.00	1.00	
Ever	0.75 (0.33–1.69)	1.38 (0.83–2.30)	
Regular laxative use[g]			0.53
Never	1.00	1.00	
Ever	1.26 (0.28–5.64)	0.73 (0.21–2.49)	
Oral antibiotic use[h]			0.46
Never	1.00	1.00	
Ever	1.00 (0.44–2.29)	0.72 (0.41–1.27)	

Period of oral antibiotic use			0.05
Childhood only (child or teenager)	0.35 (0.04–2.87)	0.27 (0.08–0.99)	
Adulthood only (age 20s or later)	1.92 (0.75–4.92)	0.55 (0.24–1.26)	
Both childhood and adulthood	0.35 (0.04–3.10)	2.06 (0.81–5.26)	
Reproductive history (females only)			
Parity[i]			0.42
0 (nulliparous)	1.00	1.00	
1–2	1.21 (0.35–4.18)	0.62 (0.28–1.39)	
≥3	0.64 (0.14–3.00)	0.21 (0.07–0.64)	
Age at first pregnancy (parous women)[i]			0.15
<30 years	1.00	1.00	
≥30 years	1.02 (0.30–3.53)	2.67 (1.16–6.14)	
Oral contraceptive use[j]			0.68
Never	1.00	1.00	
Ever	0.78 (0.25–2.43)	0.60 (0.28–1.30)	
Menopausal status[k]			0.61
Premenopausal	1.00	1.00	
Postmenopausal	2.78 (0.71–10.9)	1.94 (0.69–5.47)	

CI confidence interval; *CRC* colorectal cancer; *CT* computed tomography; *MVOR* multivariable-adjusted odds ratio; *NSAID* nonsteroidal anti-inflammatory drug.

[a]Defined based on International Classification of Diseases for Oncology, 3rd Edition (ICD-O-3) topography codes (proximal colon: C18.0, C18.2, C18.3, C18.4, and C18.5; distal colon or rectum: C18.6, C18.7, C19.9, and C20.9).

[b]MVORs were estimated from polytomous logistic regression models (CRC subsite vs. controls) adjusted for age (continuous, years), sex, family history of CRC (no, yes, unknown), regular aspirin/NSAID use (never/ever), smoking (never/ever), physical activity (active, somewhat active, insufficiently active), BMI (continuous, kg/m^2), alcohol consumption (<once/month, 1–3 times/month, 1–6 times/week, daily), red/processed meat intake (continuous, servings/week), total fruit and vegetable intake (continuous, servings/day), high-fiber food intake (continuous, servings/day), and calcium supplement use (never/ever).

[c]*p* value for heterogeneity of associations across CRC subsites, calculated using the Wald test.

[d]Among any first- or second-degree blood relative.

[e]Based only on diagnoses or medical procedures occurring at least 2 years before questionnaire completion.

[f]Includes inflammatory bowel disease (Crohn's disease and ulcerative colitis), celiac disease, rheumatoid arthritis, psoriasis, lupus, and other chronic inflammatory condition (not including allergy or asthma).

[g]Ever taken the medication regularly (at least twice per week for one month or longer) before 2 years ago.

[h]Ever used oral antibiotics repeatedly (≥2 courses per year) or for an extended period of time (>1 month) before 2 years ago.

[i]Based on pregnancies lasting for 6 months or longer. Age at pregnancy was assessed among parous women only.

[j]Ever used oral hormonal contraceptives for at least one year before 2 years ago.

[k]Females were classified as premenopausal if they had menstrual periods in the last 2 years and postmenopausal if they had stopped menstruating for at least one year before CRC diagnosis (cases) or questionnaire completion (controls) due to natural menopause or surgery to remove the uterus and/or ovaries.

Supplementary Table S5. Multivariable-adjusted odds ratios for the associations between lifestyle factors and risk of early-onset colorectal cancer according to anatomical subsite,[a] Ontario, Canada, 2018–2019

**Variables**	**Proximal colon (*n* = 41) MVOR[b] (95% CI)**	**Distal colon or rectum (*n* = 133) MVOR[b] (95% CI)**	$p_{heterogeneity}$[c]
Smoking status[d]			0.41
Never smoker	1.00	1.00	
Ever smoker	1.41 (0.67–2.97)	1.01 (0.62–1.64)	
Pack-years of smoking			0.81
Tertile 1 (<3.5)	2.72 (1.06–6.96)	1.76 (0.90–3.45)	
Tertile 2 (3.5–9.9)	0.57 (0.12–2.71)	0.63 (0.27–1.46)	
Tertile 3 (≥10.0)	1.06 (0.35–3.27)	0.76 (0.36–1.60)	
Secondhand smoke exposure[e]			
Two years ago			0.21
Never	1.00	1.00	
<2 hours/day	0.96 (0.41–2.28)	1.28 (0.73–2.25)	
≥2 hours/day	3.72 (0.96–14.4)	1.09 (0.29–4.15)	
Childhood and teenage years			0.19
Never	1.00	1.00	
<2 hours/day	0.61 (0.24–1.56)	1.55 (0.85–2.82)	
≥2 hours/day	0.96 (0.39–2.36)	1.40 (0.74–2.66)	
Alcohol consumption[f] (2 years ago)			0.13
Less than once per month	1.00	1.00	
1–3 times per month	0.41 (0.13–1.26)	1.34 (0.73–2.49)	
1–6 times per week	1.10 (0.47–2.56)	0.95 (0.53–1.70)	
Daily	1.36 (0.42–4.41)	0.99 (0.42–2.34)	
Physical activity[g] (2 years ago)			0.51
Active	1.00	1.00	
Somewhat active	1.70 (0.67–4.29)	1.06 (0.57–1.96)	
Insufficiently active	2.08 (0.91–4.71)	1.32 (0.77–2.25)	
	p_{trend}[h] = 0.08	p_{trend}[h] = 0.32	
Sedentary time[i] (2 years ago)			0.51
<5 hours/day	1.00	1.00	
5 to <10 hours/day	1.29 (0.56–2.97)	1.21 (0.70–2.09)	
≥10 hours/day	1.33 (0.44–4.05)	2.24 (1.12–4.47)	
	p_{trend}[h] = 0.58	p_{trend}[h] = 0.03	
Body mass index[j]			0.67
Two years ago			
Normal/underweight	1.00	1.00	
Overweight	0.48 (0.20–1.14)	0.61 (0.35–1.04)	
Obese	0.69 (0.30–1.62)	0.55 (0.31–1.00)	
	p_{trend}[h] = 0.46	p_{trend}[h] = 0.06	
Early age 20s			0.99
Normal/underweight	1.00	1.00	
Overweight	1.12 (0.47–2.67)	1.02 (0.58–1.82)	
Obese	0.49 (0.14–1.76)	0.43 (0.19–0.98)	

	p_{trend}[h] = 0.36	p_{trend}[h] = 0.07

CI confidence interval; *MVOR* multivariable-adjusted odds ratio.

[a]Defined based on International Classification of Diseases for Oncology, 3rd Edition (ICD-O-3) topography codes (proximal colon: C18.0, C18.2, C18.3, C18.4, and C18.5; distal colon or rectum: C18.6, C18.7, C19.9, and C20.9).

[b]MVORs were estimated from polytomous logistic regression models (CRC subsite vs. controls) adjusted for age (continuous, years), sex, family history of CRC (no, yes, unknown), regular aspirin/NSAID use (never/ever), smoking (never/ever), physical activity (active, somewhat active, insufficiently active), BMI (continuous, kg/m^2), alcohol consumption (<once/month, 1–3 times/month, 1–6 times/week, daily), red/processed meat intake (continuous, servings/week), total fruit and vegetable intake (continuous, servings/day), high-fiber food intake (continuous, servings/day), and calcium supplement use (never/ever).

[c]*p* value for heterogeneity of associations across CRC subsites, calculated using the Wald test.

[d]Ever smoked ≥100 cigarettes before 2 years ago. Pack-year was calculated by multiplying the number of packs of cigarettes smoked per day (1 pack = 20 cigarettes) by the number of years smoked.

[e]Duration of daily exposure to the tobacco smoke of others at home, work, or public places (averaged over weekdays and weekends).

[f]Frequency of drinking alcoholic beverages (e.g., 12-oz can/bottle of beer, 4-oz glass of wine, 1.5-oz shot of hard liquor) 2 years ago.

[g]Defined based on a physical activity score derived using the Godin-Shephard Leisure-Time Physical Activity Questionnaire [27], where weekly frequency (times per week) of strenuous and moderate exercise (2 years ago) was multiplied by 9 and 5, respectively, and summed across activity types to calculate the composite score (active: ≥24 units; somewhat active: 14–23 units; insufficiently active: <14 units).

[h]*p* value for linear trend calculated by treating the ordinal variable or the median value of each category (where applicable) as a continuous variable in the model, shown only when p_{trend} is < 0.10 for at least one of the subsites.

[i]Average number of hours per day spent sitting at work, at school, at home, in a car/bus/train, and during leisure time (e.g., watching TV, playing video games, using computer, reading, socializing) 2 years ago, calculated as [(5 × number of hours per day sitting on weekdays) + (2 × number of hours per day sitting on weekends)] ÷ 7.

[j]Calculated by dividing weight (kg) by height (m) squared and classified as normal/underweight (<25.0 kg/m^2), overweight (25.0–29.9 kg/m^2), or obese (≥30.0 kg/m^2).

Supplementary Table S6. Multivariable-adjusted odds ratios for the associations between dietary factors and risk of early-onset colorectal cancer according to anatomical subsite,[a] Ontario, Canada, 2018–2019

**Variables**	**Proximal colon (*n* = 41) MVOR[b] (95% CI)**	**Distal colon or rectum (*n* = 133) MVOR[b] (95% CI)**	$p_{heterogeneity}$[c]
Dietary intake 2 years ago[d]			
Total fruits and vegetables, servings/day			0.67
<3	1.00	1.00	
3 to <6	0.58 (0.25–1.31)	0.85 (0.49–1.48)	
≥6	0.44 (0.14–1.38)	0.63 (0.30–1.31)	
Fruits, servings/day[e]			0.55
<1	1.00	1.00	
1 to <3	0.68 (0.30–1.57)	1.11 (0.63–1.96)	
≥3	0.81 (0.28–2.38)	1.03 (0.49–2.15)	
Vegetables, servings/day[f]			0.45
<1	1.00	1.00	
1 to <3	0.36 (0.14–0.96)	0.63 (0.33–1.23)	
≥3	0.46 (0.15–1.44)	0.53 (0.24–1.16)	
High-fiber foods, servings/day[g]			0.53
<1	1.00	1.00	
1 to <3	1.81 (0.78–4.17)	1.09 (0.63–5.92)	
≥3	1.94 (0.63–5.92)	1.34 (0.66–2.76)	
Red meat, servings/week[h]			0.22
<2	1.00	1.00	
2–4	0.48 (0.18–1.29)	0.98 (0.51–1.90)	
≥5	0.98 (0.37–2.61)	1.05 (0.52–2.11)	
Processed meat, servings/week[h]			0.25
<1	1.00	1.00	
1–2	0.33 (0.11–1.04)	1.32 (0.62–2.80)	
≥3	1.14 (0.40–3.24)	1.25 (0.58–2.70)	
Sugary drinks, drinks/week[i]			0.07
<1	1.00	1.00	
1–6	2.33 (0.84–6.47)	1.77 (1.02–3.08)	
≥7	7.02 (2.31–21.4)	2.17 (1.07–4.39)	
	p_{trend}[j] < 0.001	p_{trend}[j] = 0.02	
Sugary desserts, times/week[k]			0.81
<3	1.00	1.00	
3–6	2.11 (0.78–5.74)	2.31 (1.24–4.30)	
≥7	1.64 (0.66–4.10)	1.37 (0.76–2.45)	
Fast food, times/week[l]			0.93
<1	1.00	1.00	
1	1.35 (0.47–3.38)	1.60 (0.83–3.07)	
≥2	1.59 (0.54–4.67)	1.99 (0.99–4.00)	
	p_{trend}[j] = 0.39	p_{trend}[j] = 0.06	

Canned food, times/week[m]			0.86
<1	1.00	1.00	
1–2	2.72 (0.93–8.00)	1.29 (0.70–2.38)	
≥3	2.35 (0.75–7.33)	1.66 (0.89–3.12)	
Processed snacks, times/day[n]			0.68
<1	1.00	1.00	
1 to <2	1.36 (0.55–3.33)	1.37 (0.78–2.40)	
≥2	2.24 (0.80–6.29)	1.38 (0.62–3.09)	
Coffee or tea, cups/day[o]			0.10
<1	1.00	1.00	
1 to <3	10.8 (1.38–84.3)	1.12 (0.61–2.06)	
≥3	10.9 (1.31–91.1)	1.39 (0.68–2.86)	
	p_{trend}[j] = 0.02	p_{trend}[j] = 0.39	
Water, glasses/day			0.34
<3	1.00	1.00	
3 to <8	0.77 (0.35–1.67)	1.15 (0.68–1.93)	
≥8	1.19 (0.40–3.47)	0.78 (0.35–1.71)	
Artificial sweeteners, times/week			0.40
<1	1.00	1.00	
1–6	1.86 (0.78–4.42)	1.00 (0.53–1.87)	
≥7	1.63 (0.56–4.75)	1.72 (0.88–3.38)	
Agave syrup, times/week			0.20
<1	1.00	1.00	
≥1	0.13 (0.02–1.16)	0.58 (0.24–1.42)	
Western-like dietary pattern score[p]			0.45
Quartile 1 (0–9)	1.00	1.00	
Quartile 2 (10–13)	1.54 (0.51–4.62)	1.45 (0.75–2.78)	
Quartile 3 (14–17)	1.77 (0.57–5.49)	1.57 (0.80–3.09)	
Quartile 4 (18–27)	3.31 (1.10–9.94)	1.54 (0.76–3.13)	
	p_{trend}[j] = 0.03	p_{trend}[j] = 0.21	
Supplement use before 2 years ago[q]			
Calcium supplement			0.06
Never	1.00	1.00	
Ever	1.01 (0.43–2.37)	0.40 (0.22–0.76)	
Antacid			0.003
Never	1.00	1.00	
Ever	2.20 (1.04–4.63)	0.64 (0.39–1.06)	
Vitamin D/cod liver oil supplement			0.65
Never	1.00	1.00	
Ever	0.97 (0.45–2.09)	0.81 (0.49–1.34)	
Prebiotic fiber supplement			0.29
Never	1.00	1.00	
Ever	1.52 (0.66–3.48)	0.93 (0.51–1.71)	
Probiotic supplement			0.34
Never	1.00	1.00	
Ever	0.74 (0.27–2.01)	1.24 (0.68–2.25)	

Folic acid supplement (females only)			0.42
Never	1.00	1.00	
Ever	1.16 (0.37–3.70)	0.71 (0.34–1.47)	

CI confidence interval; *MVOR* multivariable-adjusted odds ratio.

[a]Defined based on International Classification of Diseases for Oncology, 3rd Edition (ICD-O-3) topography codes (proximal colon: C18.0, C18.2, C18.3, C18.4, and C18.5; distal colon or rectum: C18.6, C18.7, C19.9, and C20.9).

[b]MVORs were estimated from polytomous logistic regression models (CRC subsite vs. controls) adjusted for age (continuous, years), sex, family history of CRC (no, yes, unknown), regular aspirin/NSAID use (never/ever), smoking (never/ever), physical activity (active, somewhat active, insufficiently active), BMI (continuous, kg/m^2), alcohol consumption (<once/month, 1–3 times/month, 1–6 times/week, daily), red/processed meat intake (continuous, servings/week), total fruit and vegetable intake (continuous, servings/day), high-fiber food intake (continuous, servings/day), and calcium supplement use (never/ever).

[c]*p* value for heterogeneity of associations across CRC subsites, calculated using the Wald test.

[d]All food and beverage variables are based on usual consumption 2 years before questionnaire completion.

[e]Examples for one serving of fruit: 1 medium-sized fresh fruit, 1/2 cup of chopped, cooked, or canned fruit, 1/4 cup of dried fruit, 1/2 cup of fruit juice.

[f]Examples for one serving of vegetables: 1 cup of raw leafy vegetables, 1/2 cup of other vegetables (cooked, canned, frozen, or chopped raw), 1/2 cup of vegetable juice.

[g]Foods high in fiber, such as wholegrain bread (not white bread), wholegrain or high-fiber breakfast cereal/muesli/bran, brown rice, barley, oats, and legumes (beans, peas, lentils). Examples of one serving of high-fiber food: 1 slice of wholegrain bread, 1/2 cup of cooked or cold high-fiber cereals, 1/2 cup of brown rice or wholegrains.

[h]One serving of red/processed meat defined as 2–3 oz or the size of the palm of hand.

[i]Sugary drinks such as soft drinks (excluding diet soda), vitamin drinks, energy drinks, and specialty coffee with syrup (e.g., mocha).

[j]*p* value for linear trend calculated by treating the median value of each category as a continuous variable in the model, shown only when p_{trend} is < 0.10 for at least one of the subsites.

[k]Desserts containing sugar, such as candy, chocolate bars, cake, cookies, and ice cream.

[l]Includes foods from fast food restaurants (e.g., burger, fries, taco), pizza, and instant meals (e.g., instant ramen noodles).

[m]Any canned foods (e.g., canned corn, canned fruit, canned tomato sauce).

[n]Any processed snack foods such as chips, crackers, white bread, and sugary cereals.

[o]Includes both caffeinated and decaffeinated coffee or tea. Estimates may not be reliable for proximal colon cancer due to small cell sizes.

[p]Composite dietary score derived based on 6 non-beneficial (red meat, processed meat, sugary drinks, sugary desserts, fast food, and processed snacks) and 3 beneficial (fruits, vegetables, and high-fiber foods) components. For each non-beneficial component, subjects in the first, second, third, and fourth quartile of intake were assigned a value of 0, 1, 2, and 3, respectively; for each beneficial component, quartiles were reverse coded (i.e., 3, 2, 1, and 0, respectively). The final score was calculated by summing up values across all 9 components, with higher scores indicating a more Westernized dietary pattern. The MVORs were adjusted for all variables listed in footnote b, except for red/processed meat, total fruit and vegetable, and high-fiber food intake.

[q]Ever used the supplement regularly (at least once per week for at least one month) before 2 years ago.
